# Case report: Pediatric intraventricular Rosai-Dorfman disease: clinical insights and surgical strategies in a decade-long observational study and literature review

**DOI:** 10.3389/fonc.2024.1487835

**Published:** 2024-11-29

**Authors:** Dayuan Liu, Ning Li, Yubo Zhu, Yunxiang Zhong, Guolong Deng, Mingfa Wang, Caicai Zhang, Jigao Feng

**Affiliations:** ^1^ Department of Neurosurgery, The Second Affiliated Hospital of Hainan Medical University, Haikou, Hainan, China; ^2^ Department of Pathology, The Second Affiliated Hospital of Hainan Medical University, Haikou, Hainan, China; ^3^ Department of Physiology, Hainan Medical University, Haikou, Hainan, China; ^4^ Department of Surgery, Hainan Vocational University of Science and Technology, Haikou, Hainan, China

**Keywords:** Rosai-Dorfman disease (RDD), intraventricular tumor, pediatric epilepsy, central nervous system histiocytosis, surgery

## Abstract

**Background:**

Rosai-Dorfman disease (RDD), or sinus histiocytosis with massive lymphadenopathy (SHML), is a rare benign disorder characterized by the proliferation of histiocytes of uncertain origin. Central nervous system (CNS) involvement, particularly intraventricular, is exceptionally rare and poses significant diagnostic challenges due to its non-specific clinical and radiographic presentation. This study aims to present a case of intraventricular RDD and review existing literature on its clinical features, treatment strategies, and prognosis.

**Methods:**

We report the case of a five-year-old male with recurrent headaches and epilepsy caused by an intraventricular mass. The mass was surgically resected and histopathological examination was performed to confirm the diagnosis. A comprehensive literature review was conducted to identify similar cases of intraventricular RDD, focusing on clinical features, diagnostic methods, treatment strategies, and outcomes.

**Results:**

Histopathological examination of the resected tumor revealed typical features of RDD, including large histiocytes, lymphocyte infiltration, and immunohistochemical positivity for CD68, S-100, and Vimentin. The patient remained asymptomatic ten years post-surgery with no recurrence of epilepsy or tumor. The literature review identified six similar cases, all of which showed favorable outcomes post-surgery, highlighting the self-limiting nature and favorable prognosis of intraventricular RDD following surgical resection.

**Conclusion:**

Intraventricular RDD, though rare, should be considered in the differential diagnosis of intraventricular masses in pediatric patients. Surgical resection remains the primary treatment modality, and histopathological confirmation is essential for accurate diagnosis. The prognosis is generally favorable with appropriate surgical intervention, although recurrence can occur, necessitating long-term follow-up. Further research is required to refine diagnostic criteria and explore adjuvant therapies for improved management of this rare CNS disorder.

## 1 Introduction

Sinus histiocytosis with massive lymphadenopathy (SHML), also known as Rosai-Dorfman disease (RDD), is a rare, benign disorder characterized by proliferative histiocytic activity of uncertain origin. Initially considered a non-neoplastic reactive histiocytic disease linked to autoimmunity (8), recent research has suggested an infectious theory, with some scholars proposing associations with specific infections such as cytomegalovirus, parvovirus B19, and Epstein-Barr virus (12, 14).

First described in 1969 by Rosai and Dorfman, the term RDD originated from their observation of four cases that demonstrated distinctive clinical presentations and histopathological features of histiocytic proliferative disease (1). The hallmark of RDD is the infiltration of sinus histiocytes in lymph nodes, typically presenting as painless lymphadenopathy (12, 14). Central nervous system (CNS)-RDD, particularly within ventricular systems, is exceptionally rare in clinical literature, with only a limited number of reported cases globally (3). Due to its low incidence rate and varied affected regions with associated pathological changes, alongside non-specific radiographic findings on CT and MRI scans, the potential for misdiagnosis or delayed diagnosis before definitive histopathological examination is considerable.

The disease predominantly affects children and young adults, with a peak incidence at around 20 years of age, and shows a higher prevalence in males than females. Related key manifestations include painless cervical lymphadenopathy, fever, weight loss, and non-specific symptoms resembling infection, such as elevated erythrocyte sedimentation rate (14). Some cases present with extranodal involvement without lymphadenopathy, affecting various organs, including the skin, nasal cavity, sinuses, eyelids, orbits, bones, and digestive system (11). In fewer than 5% of RDD cases, the disease involves the CNS, typically affecting sites like the suprasellar region, cerebral convexity, parasagittal region, cavernous sinus, and petroclival region. The most common CNS manifestation resembles a dural mass similar to meningioma (16, 23), and intraventricular involvement is exceptionally rare.

In the 2016 WHO classification of CNS tumors, RDD was categorized as a histiocytic tumor characterized by the infiltrative growth of non-neoplastic histiocytes in lymph nodes and extranodal sites (11). CNS involvement in RDD, termed CNS-RDD, is more frequently observed in older patients, with lesions typically found in locations such as the cerebellopontine angle, sellar region, and occipital lobe (5, 10). Clinical symptoms of CNS-RDD are non-specific and vary depending on the lesion’s location, size, and impact on adjacent nerve function. Common symptoms include headaches, limb paralysis, sensory disturbances, and other neurological deficits. Lesions in the sellar region can lead to visual impairment and pituitary dysfunction similar to those seen in pituitary adenomas, while involvement of the ventricular system may result in obstructive hydrocephalus, potentially leading to coma and even death (15). RDD originating within the ventricles is rare, with only a few documented cases in the literature to date (6).

Currently, there is a lack of specific diagnostic methods for CNS-RDD. Due to its non-specific imaging characteristics, absence of distinctive histological features, or potential overlap with other concurrent lesions, RDD is frequently misdiagnosed as a tumor. Clinical cases often mimic meningiomas due to their solitary mass appearance, contributing to misdiagnosis in more than 90% of cases (9). The challenge of diagnosing RDD escalates when it presents as solitary lesions within the cerebral ventricles, as it lacks the typical features of dural-based lesions associated with this condition.

The aim of this study is to enhance understanding on intraventricular RDD by presenting a detailed case report and conducting a literature review, detailing the clinical manifestations, diagnostic challenges, treatment modalities and long-term outcomes associated with this rare CNS disorder, thereby aiding clinicians in recognizing and managing similar cases effectively.

## 2 Case description and methods

### 2.1 Patient profile

A five-year-old male child was admitted to our hospital on March 11, 2011, due to a primary complaint of recurring headaches and convulsions in the extremities that had persisted for over three years and intensified one day prior to admission. Before this period, the child had experienced episodic headaches accompanied by limb rigidity and convulsions lasting approximately ten seconds before spontaneous resolution. Previously evaluated at a local hospital, an electroencephalogram (EEG) showed no significant abnormalities, while a cranial MRI revealed a space-occupying lesion in the right cerebral ventricle. Initial treatment with anti-epileptic medications resulted in symptom improvement, leading to discharge. However, the day before this admission, the child experienced a sudden onset of headache and vomiting, necessitating further evaluation at our facility. The patient had no significant familial medical history.

Upon admission, the child was conscious and responsive, with a Glasgow Coma Scale (GCS) score of E4V5M6 = 15. His cranial examination showed a normal-sized cranium with even hair distribution. Pupillary examination revealed equal and round bilateral pupils of approximately 3.0mm in diameter, responsive to light. No nystagmus or mouth angle deviation was observed, there was no neck resistance, and muscle strength and tone in all four limbs were normal. Pathological reflexes were not present.

### 2.2 Diagnostic investigation

**Figure 1:**
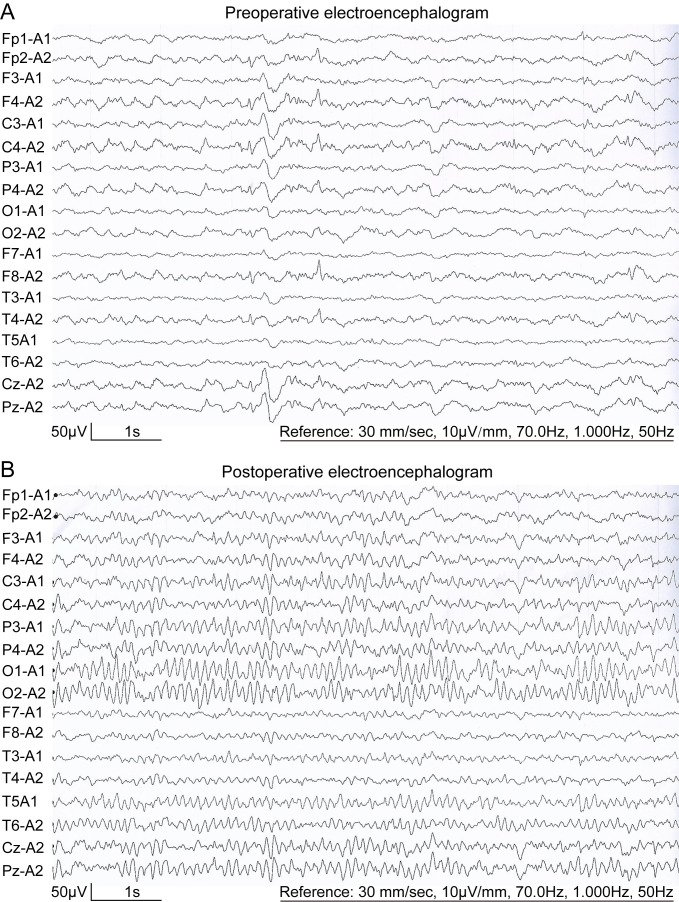
Eighteen-lead resting-state EEG of the patient. **(A)** Preoperative EEG of the patient showed sporadic spikes/sharp waves in the frontal and temporal lobes. **(B)** Postoperative EEG showed a normal EEG.

**Figure 2:**
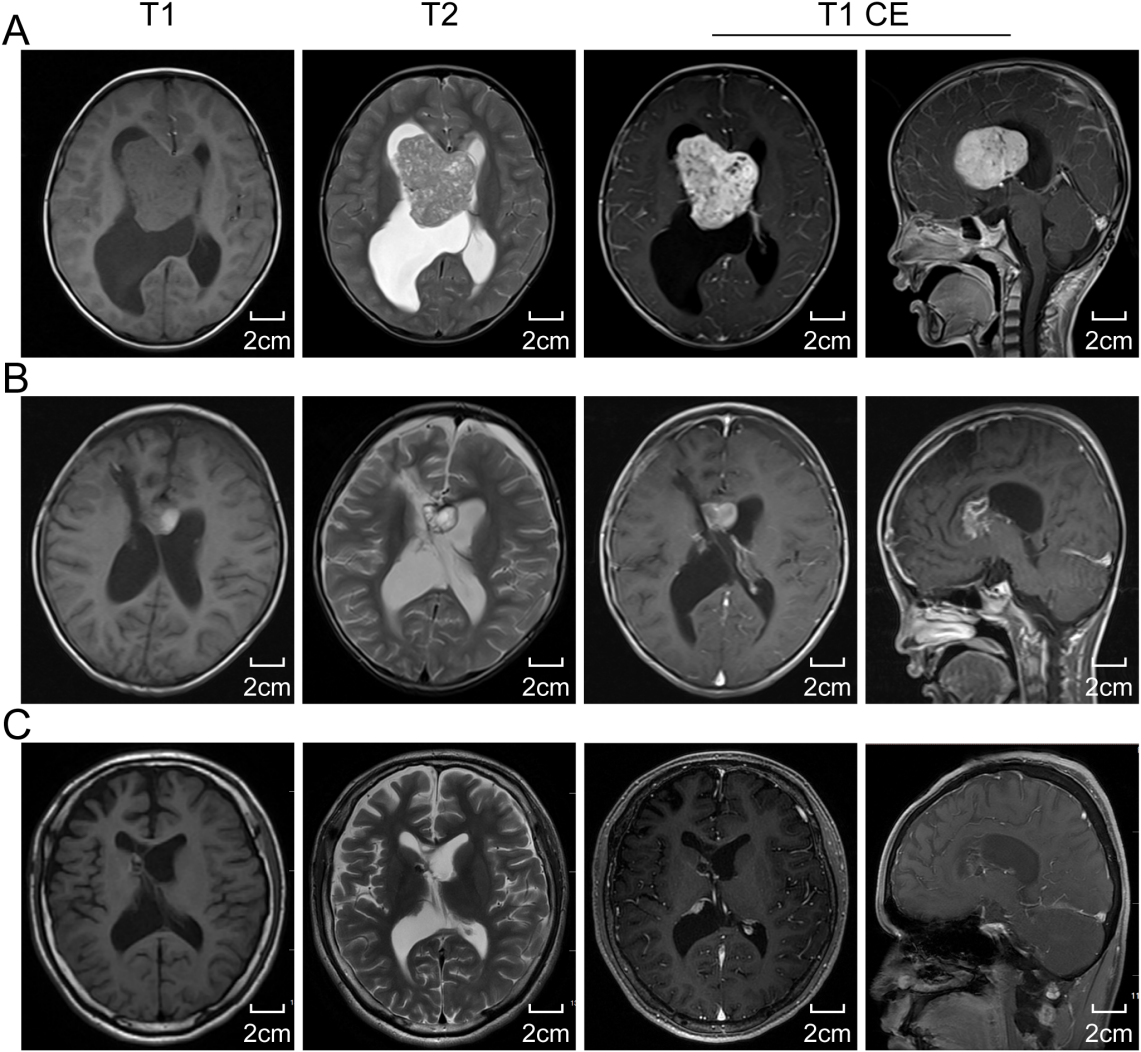
The cranial magnetic resonance imaging (MRI) results. **(A)** Preoperative standard MRI reveals a lesion within the lateral ventricle. The lesion presents as isointense with gray matter on T1-weighted images and hyperintense on T2-weighted images. Axial and sagittal views of enhanced T1-weighted images display uniformly enhanced intraventricular lesion, with obstructive hydrocephalus due to a blockage at the Monro foramen. **(B)** A two-week postoperative MRI follow-up suggests a subtotal resection of the tumor, with nodular enhancement indicating residual tumor within the ventricle at the genu of the corpus callosum. **(C)** A ten-year postoperative MRI follow-up indicates near disappearance of the intraventricular tumor and significant relief of hydrocephalus. T1 CE, T1-weighted contrast-enhanced imaging.

The EEG showed mild abnormal electrical activity (**Figure 1A**), which normalized post-surgery (**Figure 1B**). Cranial MRI revealed a well-defined mass measuring approximately 59×50×46mm^3^ in the anterior region of the right ventricle. The mass had clear margins, a lobulated appearance, low signal intensity on T1-weighted images, mixed high signal intensity on T2-weighted images, and significant enhancement with contrast. Bilateral lateral ventricles are dilated, especially the right ventricle, and high signals can be seen around the anterior horn of the lateral ventricle on T2-weighted images (**Figure 2A**). The results of blood tests, urinalysis and other investigations were within normal limits. Based on these, the preliminary diagnosis was right lateral ventricular tumor with obstructive hydrocephalus and secondary epilepsy.

### 2.3 Therapeutic intervention

A right frontal parietal craniotomy was conducted under general anesthesia with endotracheal intubation to resect the intraventricular tumor. Intraoperatively, the tumor was found to have a soft, purplish-red appearance, distinct from the surrounding brain tissues, and was encapsulated, measuring approximately 6cm×6cm. It was resected in sequential blocks, ensuring careful preservation of vasculature and avoiding venous injury. The excised tissue was then submitted for histopathological examination.

### 2.4 Literature assessment

A comprehensive search was conducted using databases such as PubMed, MEDLINE, and Scopus to perform the literature review. The keywords used included “Rosai-Dorfman Disease”, “intraventricular”, “central nervous system”, and “pediatric.” The study inclusion criteria encompassed case reports, clinical studies, and reviews published in peer-reviewed journals. Briefly, articles were screened for relevance based on the abstract, and full texts were analyzed to extract data on clinical features, diagnostic methods, treatment strategies, and outcomes of intraventricular RDD. Data synthesis and analysis focused on identifying patterns in presentation, management, and prognosis.

### 2.5 Statistical analysis

Descriptive statistics summarized patient demographics (age, gender, clinical presentation), tumor characteristics (location, size, MRI findings), treatment approaches (surgical methods, adjuvant therapies), and outcomes (follow-up duration, recurrence rates, symptom resolution).

## 3 Results

### 3.1 Postoperative pathology

**Figure 3:**
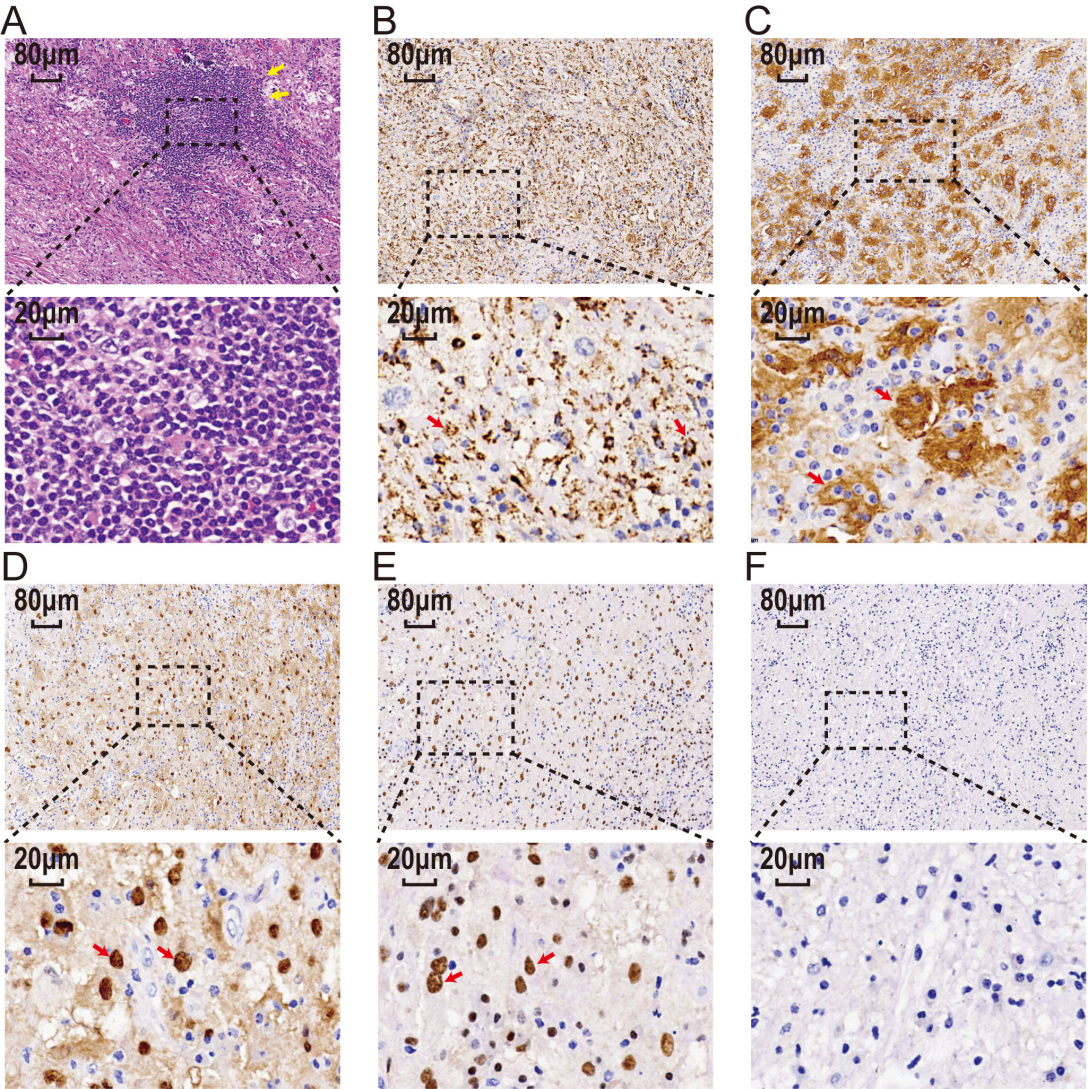
The histopathological examination results. **(A)** Hematoxylin-eosin stain of tissue sections shows atypical histiocytic cells with emperipolesis(yellow arrow). Large histicytes immunoreactive for CD68 **(B)**, S100 **(C)**, CyclinD1 **(D)**, and OCT2 **(E)** (red arrow) and immunonegatie for CD1a **(F)**. Scale bar: 20 μm and 80 μm. CD68, Cluster of Differentiation 68; S100, S100 protein; OCT2, Organic Cation Transporter 2; CD1a, Cluster of Differentiation 1a.

The postoperative histopathological examination revealed a lesion composed of large histiocytes with round nuclei and prominent nucleoli. The lesion showed infiltration of lymphocytes and plasma cells, along with perivascular cuffing of lymphocytes in localized vascular areas. Immunohistochemical staining showed positivity for CD68, S-100, and Vimentin, partial positivity for GEAP, and a low Ki-67 proliferation index (<10%). CD1a staining was negative. Based on these findings, the lesion was diagnosed as consistent with RDD (**Figures 3A–F**).

### 3.2 Postoperative follow-up

A follow-up MRI performed two weeks post-surgery demonstrated near-total resection of the lesion, along with a low-signal lesion in the right frontal lobe, indicative of post-surgical changes, and evidence of encephalomalacia. Ventricular enlargement and cerebral edema showed improvement compared to previous scans (**Figure 2B**). A residual tumor was noted as a nodular enhancement at the genu of the corpus callosum on the right ventricle wall.

The patient did not receive postoperative radiotherapy or adjuvant therapy. Ten years post-surgery, the patient remained asymptomatic with no headaches or dizziness, and no recurrence of epilepsy. Follow-up cranial MRI revealed a reduction in the size of the nodular enhancement at the genu of the corpus callosum on the right ventricle wall compared to earlier scans (**Figure 2C**). There was no radiographic evidence of tumor recurrence. The patient’s growth and development were normal, and follow-up continued via telephonic consultations.

### 3.3 Literature review of RDD

Next, we summarized the clinical features of our case and reviewed other cases of intraventricular RDD (**Table 1**). As can be seen, each case showed pathological abnormalities mainly localized within the cerebral ventricles, affecting both the lateral and fourth ventricles, either as solitary or multifocal lesions (8).

**Table 1:** Summary of the clinical features of cases with intraventricular Rosai-Dorfman disease.

Year/study	Gender/Age	Location	Clinical presentation	Surgery	Adjuvant therapy	Outcome	Follow-up
1998 (2)	F/40	Right lateral ventricle; Single lesion	Headache	Total resection	No	Asymptomatic	unknown
2015 (1)	F/2	Left lateral ventricle; Single lesion	Vomiting, fever	Subtotal resection	No	Asymptomatic	16months
2021 (15)	F/9	Bilateral lateral ventricles; Multiple lesions	Blurred vision, facial paralysis, giggle and cognitive impairment	Total resection	No	Asymptomatic	15 months
2022 (16)	M/8	Bilateral lateral ventricles; Multiple lesions	Nausea, vomiting and ataxia	Total resection	Radiotherapy/ chemotherapy	Asymptomatic	12 months
2021 (7)	M/30	The fourth ventricle; Single lesion	Vomiting, weight loss, dysphagia and vertigo	Resection	Chemotherapy	Remission	unknown
2024/This study	M/5	Right lateral ventricles; Single lesion	Seizures, headache	Subtotal resection	No	Asymptomatic	120 months

Specifically, four cases involved a single intraventricular lesion, while the other two cases featured multiple lesion sites. According to the literature, most patients initially presented with symptoms indicative of increased intracranial pressure, such as headache and vomiting. Some also exhibited cognitive impairment and ataxia, depending on the locations invaded by the tumor. However, cases of ventricular RDD complicated by seizures were rare. In comparison with a case involving a child with RDD in the right ventricle and a lobulated enhancing mass in the fourth ventricle, our patient was slightly younger. They presented symptoms of supratentorial ventricular enlargement and obstructive hydrocephalus.

Clinical symptoms primarily included headache and dizziness, along with secondary epilepsy symptoms such as limb tetanic convulsions. These symptoms may be attributed to physiological blockage of cerebrospinal fluid circulation by the right ventricular tumor, leading to increased intracranial pressure, reduced cerebral blood flow perfusion causing cerebral ischemia and hypoxia, and subsequent epilepsy. Alternatively, compression of the frontal/temporal lobe by the ventricular tumor could induce corresponding lobar epilepsy symptoms.

In our present case report, the patient did not have hepatosplenomegaly or other manifestations of extranodal lymph node diseases, suggesting it was an isolated intraventricular presentation of RDD, which is rare. Surgical treatment was performed on all six reported patients, and despite varying initial conditions and extents of tumor resection, most patients showed favorable outcomes. Compared to the literature review, additional benefits were observed in some patients who received post-surgical radiotherapy or steroid therapy.

## 4 Discussion

Our study presents a rare case of pediatric intraventricular RDD associated with epilepsy, managed successfully through surgical intervention. This case, along with five others documented in the literature, emphasizes the self-limiting nature of intraventricular RDD and its favorable prognosis following subtotal or total resection. Notably, all patients demonstrated significant clinical improvement post-surgery, with no recurrence or malignant progression observed during follow-up periods extending up to ten years.

The head MRI of our presented case revealed a lesion in the right ventricle characterized by irregular lobulated enhancement with well-defined borders, accompanied by ventricular enlargement and hydrocephalus. Initially, the clinical diagnosis considered choroid plexus papilloma, with meningioma also under consideration. However, unlike most choroid plexus papillomas, intracranial RDD typically shows high or isointense signals on T1-weighted MRI with clear boundaries. T2-weighted imaging usually displays isointense signals, with areas of low signal intensity that enhance uniformly post-contrast. Some researchers suggest that these low-signal areas could result from free radicals produced during macrophage phagocytosis activity (19). However, such a phenomenon was not observed in our case, possibly due to the location of the lesion within the ventricles and inadequate lymphocyte aggregation. Therefore, differentiating RDD from other intraventricular tumors in adolescents, such as choroid plexus papillomas, ependymomas, subependymal giant cell astrocytomas, and central neurocytomas, is essential, and accurately diagnosing isolated intraventricular RDD remains a significant challenge for clinicians.

Recently, the new MRI sequences recommended for diagnosing RDD include diffusion tensor imaging, magnetic resonance susceptibility weighted imaging, and perfusion-weighted imaging (20). Meningiomas are characterized by a rich blood supply, whereas RDD shows varying degrees of vascularity (22). Therefore, magnetic resonance angiography (MRA), magnetic resonance venography (MRV), or angiography can aid in distinguishing between RDD and meningioma. However, due to the non-specific nature of clinical auxiliary examinations, accurately assessing the nature of the lesion based solely on clinical manifestations and imaging is extremely challenging, especially without prior tissue biopsy. Thus, histological diagnosis remains the gold standard for confirming RDD.

According to the extent of lesion involvement, RDD can be pathologically classified into three subtypes: nodal type, extranodal type, and mixed type involving both lymph nodes and extranodal organs (11). Low-power microscopic examination of pathological sections often reveals nodules of varying sizes with alternating pale and deeply stained areas. Eosinophilic granulocytes are rare, and histiocytes phagocytizing inflammatory cells, a phenomenon known as “emperipolesis”, represent a typical pathological sign of RDD. However, about 35% of cases do not exhibit this feature due to extensive fibrous tissue and inflammatory cells obscuring typical histiocytic morphology (2, 13). When “emperipolesis” is not prominent, the diagnosis of RDD relies on specific immunohistochemical staining patterns: strong positive expression of S-100 protein and CD68, and negative expression of CD1a and GFAP in RDD histiocytes (24). The presence of inflammatory cells, predominantly composed of lymphocytes, further supports the diagnosis. Cases diagnosed as RDD on histopathology must also be differentiated from conditions such as Langerhans cell histiocytosis, lymphoplasmacyte-rich meningioma, malignant fibrous histiocytoma, chronic non-specific inflammation, and more (4). Additionally, distinguishing primary ventricular RDD from common childhood ventricular tumors like ependymoma, astrocytoma, choroid plexus papilloma, teratoma, and meningioma is crucial. In our case, presenting with headache, dizziness, and epilepsy symptoms, histopathological examination revealed sinus tissue proliferation resembling giant lymph node disease. Immunohistochemistry demonstrated strong positive staining for S-100 protein and CD68. Based on tissue morphology and immunohistochemical staining results, RDD in the ventricle was confirmed.

Due to the rarity of primary intracranial RDD, particularly ventricular RDD complicated by epilepsy, there are currently no established treatment guidelines. Surgical resection remains widely recognized as an effective therapeutic approach by most experts. Post-surgical pathological diagnosis provides crucial guidance for potential adjuvant therapies. RDD is classified as a non-neoplastic disorder, and patients who undergo complete resection generally have better prognoses. However, there is a risk of recurrence post-surgery, often correlated with the extent of resection. Therefore, complete lesion removal is recommended, accompanied by long-term patient follow-up. In recent years, some researchers have suggested that in cases where surgical resection is not feasible or as an adjunct to surgery, low-dose radiotherapy, hormone therapy, immunosuppressive therapy, or antiviral therapy may offer therapeutic benefits for RDD. However, such treatments are based on limited reported cases on CNS-RDD, necessitating further clinical validation and experience to establish their efficacy (18, 21). Moreover innovative advancements in the comprehension of molecular disturbances associated with RDD have furnished fresh insights for its therapeutic interventions (17). Specifically, alterations in the B-Raf Proto-Oncogene, Serine/Threonine Kinase (BRAF), mitogen-activated protein kinase (MAPK), A-Raf Proto-Oncogene, Serine/Threonine Kinase (ARAF), and Rat Sarcoma Viral Oncogene Homolog (RAS) pathways have been identified in RDD, presenting potential therapeutic targets (6). Several studies have demonstrated the responsiveness of RDD lesions to MAPK inhibitors, suggesting that targeting the MAPK pathways could offer a novel therapeutic strategy for RDD (7). However, further research is necessary to validate these targets in clinical trials and to explore other potential therapeutic targets within RDD.

In conclusion, this case report and literature review highlight the importance of considering RDD in the differential diagnosis of intraventricular tumors in pediatric patients. The favorable prognosis observed in intraventricular RDD cases suggests that individualized surgical interventions and vigilant postoperative monitoring can lead to excellent long-term outcomes. Future research could focus on refining diagnostic criteria and exploring potential adjuvant therapies to further improve management strategies for this rare but treatable condition.

## Data Availability

The original contributions presented in the study are included in the article/supplementary material. Further inquiries can be directed to the corresponding authors.
